# Developing and validating entrustable professional activities for neurology residency training programs in Saudi Arabia: a modified Delphi study

**DOI:** 10.3389/fneur.2025.1688620

**Published:** 2025-12-02

**Authors:** Amal M. Alkhotani, Abdullah Tawakul, Laila A. Alharbi, Manal E. Alotaibi, Mohammed S. Samannodi, Hussam M. Alim, Hadeel A. Khadawardi, Fahd Almalki, Ahmad A. Imam, Mohammad S. Dairi, Yosra A. Turkistani, Adeeb A. Bulkhi, Rania Zaini, Hani M. Almoallim

**Affiliations:** 1 Department of Medicine, College of Medicine, Umm Al-Qura University, Makkah, Saudi Arabia; 2 Department of Community Medicine and Pilgrims Healthcare, Umm Al-Qura University (UQU), Makkah, Saudi Arabia

**Keywords:** entrustable professional activities, EPAS, neurology, Saudi Arabia, curriculum, residency education, professional tasks, expert consensus

## Abstract

**Background and objective:**

Entrustable professional activities (EPAs) are tasks that medical professionals can be entrusted to perform in unsupervised settings once they have achieved sufficient specific competencies. Despite their importance, nationally validated neurology EPAs are lacking in Saudi Arabia. This study aimed to develop and validate neurology EPAs in Saudi Arabia.

**Methods:**

A list of neurology EPAs was developed after an extensive review of existing neurology training program competencies and EPAs. Neurology experts were invited to participate in two rounds of a modified Delphi technique to review the list of EPAs and assess their relevance and representativeness using a 5-point Likert scale. In total, 21 neurologists participated in the study. Descriptive statistics were used to describe participants’ demographic characteristics and group responses to each EPA. Cronbach’s alpha was used to assess the internal consistency of the responses across both Delphi rounds.

**Results:**

In the first round, 26 EPAs were validated, 10 were excluded owing to a lack of relevance, and one EPA was added as a modification of the existing items. In the second round, one more EPA was excluded because of a lack of relevance, resulting in a final set of 25 neurology EPAs.

**Conclusion:**

This study developed and content-validated a set of EPAs for neurology residency training in Saudi Arabia. It represents an initial step toward implementing an EPA-based curriculum. Further steps are necessary to ensure adequate integration into training programs.

## Graphical abstract

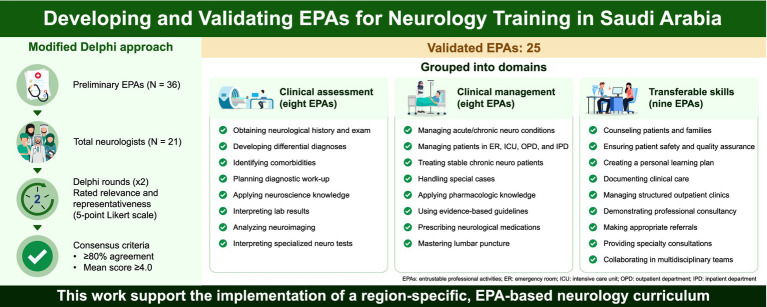

## 1 Introduction

Entrustable professional activities (EPAs) are tasks that medical professionals can be entrusted to perform in unsupervised settings once they have achieved sufficient specific competencies (1). EPAs integrate multiple competencies or milestones in real clinical settings and reflect tasks essential for safe and effective practice (2). These activities result from the accumulation of knowledge and skills related to specific specialties. Since their introduction in 2005 (3), EPAs have increasingly been used to define healthcare curricula (4), and EPA-based curricula have been implemented across different stages of medical education (5–12).

In recent years, several specialties, such as internal medicine, anesthesiology, psychiatry, pediatrics, orthopedics, physical medicine, and rehabilitation, have established EPA frameworks to better align training outcomes with real-world clinical practice (6, 8, 13–22). In 2020, the Royal College of Physicians and Surgeons of Canada adopted neurology EPAs within a competency-based framework (22). Furthermore, a Singaporean group developed five neurology EPAs for workplace assessment (23), although the results of their study have not yet been published. Despite these advances, structured EPA development in neurology has received relatively limited attention, particularly in Saudi Arabia.

The neurology residency program in Saudi Arabia was established in 1999 under the Saudi Commission for Health Specialties (SCFHS). Initially designed as a 4-year program, it was extended to 5 years in 2016 to better accommodate evolving training needs (24). The program aims to graduate competent clinical neurologists, with core aspects of training including interpersonal and communication skills, professional development, ethical conduct, integration of evidence-based medicine into clinical practice, and quality assurance. The program is competency-based, with specific competencies defined according to the level of training and clinical rotations (24).

Competency-based frameworks provide valuable guidance for learners, supervisors, and institutions regarding teaching and assessment (25). Despite the emphasis on competencies, their integration into the clinical practice of graduating physicians remains limited (25). Competencies describe the ability of the individual to perform specific tasks, whereas EPAs specify the professional activities that can be entrusted to the trainee (25).

Given the variation in healthcare systems and educational structures across countries, adopting EPA models developed elsewhere may not align with local practices. Therefore, developing EPAs specific to national or regional contexts is essential. A large project aimed at developing and validating EPAs across several internal medicine fellowship programs in Saudi Arabia is underway, with a few publications available to date (17–19). As part of this project, the present study aimed to develop and validate end-of-training EPAs for neurology in Saudi Arabia.

## 2 Materials and methods

This study aimed to develop and validate a consensus-based list of EPAs for neurology residency programs in Saudi Arabia, using a modified Delphi technique. The study was conducted in two phases: a preparatory phase involving expert review and EPA generation, followed by two rounds of a modified Delphi technique.

### 2.1 Standard protocol approvals, registrations, and patient consents

**Figure 1:**
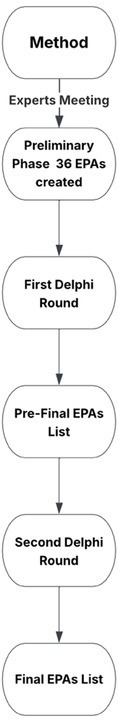
Design and flow of the study.

Ethical approval was obtained from the Institutional Review Board of Umm Al-Qura University, Makkah, Saudi Arabia (IRB approval number HAPO-02-K-012-2024-04-2098). The study complies with the Declaration of Helsinki. Informed consent was obtained from all participants. The study design and flow are illustrated in Figure 1.

The target participants comprised board-certified neurologists practicing in Saudi Arabia with a minimum of 3 years of clinical experience and active involvement in postgraduate neurology training programs; those with exactly 3 years of experience were also eligible if they had at least 2 years of active engagement as a trainer in postgraduate training. Participants were recruited using purposive sampling to ensure diversity across institutional affiliations and geographic regions. In total, 45 expert neurologists were invited, each receiving an orientation package outlining the study’s objectives, methodology, and expectations.

### 2.2 Phase 1: development of preliminary EPA list

In the preliminary phase, a team of two senior neurologists and two medical education experts conducted a thorough review of national and international neurology training competencies, including the SCFHS neurology training objectives, Royal College of Physicians and Surgeons of Canada neurology residency competencies and related EPAs, and the Neurology Core Competencies Outline of the American Board of Psychiatry & Neurology (22, 24, 26, 27). The senior neurologist had more than 10 years of experience and active participation in postgraduate training. Based on this review, a list of 36 preliminary neurology EPAs was generated and categorized into four domains: clinical assessment, clinical management, procedures, and transferable skills. This list served as the foundation for the modified Delphi rounds.

### 2.3 Phase 2: Delphi rounds

A two-round modified Delphi technique was employed to refine and validate the proposed EPAs. The Delphi panel included experts who met the eligibility criteria and agreed to participate. Demographic data, including years of experience in neurology and training, were collected prior to the first round.

In the first round, participants rated each of the 36 preliminary EPAs for relevance and representativeness on a 5-point Likert scale (1 = not important/relevant, 5 = very important/relevant). In addition to quantitative ratings, participants were invited to provide written qualitative feedback on each EPA. These comments were systematically reviewed by the research team to identify ambiguities, contextual limitations, or suggestions for modification. EPAs that did not reach consensus but received constructive feedback were revised and rephrased for reconsideration in the second round rather than being excluded outright. This combined analysis of quantitative ratings and qualitative feedback ensured that the final EPA list reflected both statistical consensus and expert insights.

Based on the results of the first round, 10 EPAs with a mean score <4.0 or <80% agreement (defined as the proportion of panelists rating the EPA as 4 or 5) were excluded, and one EPA was modified based on expert comments. The revised list was then carried forward to the second round.

In the second round, the same panel of experts reviewed the revised list of EPAs and reassessed their relevance and representativeness using the same Likert scale. The aim of this round was to confirm consensus on retained EPAs and to assess agreement with the modifications made following the first round. Panelists were given the opportunity to provide further comments on the pre-final list and were asked to indicate their agreement with the introduced modifications.

### 2.4 Data analysis

Quantitative data from each Delphi round were analyzed using descriptive statistics, including the mean, standard deviation, and percentage agreement. Consensus was defined as a mean rating ≥4.0, with at least 80% of panelists rating the EPA as 4 or 5. Although Likert data are ordinal, using mean scores and percentage agreement thresholds is widely accepted in Delphi studies, particularly in EPA development research, to operationalize consensus and enable direct comparison across rounds (17–20). This approach was therefore adopted to maintain methodological consistency with previous national Delphi studies and to facilitate interpretation of results.

EPAs that did not meet the consensus threshold were either excluded or reconsidered for modification if supported by constructive qualitative feedback. Qualitative feedback provided by panelists in free-text form was analyzed thematically by four authors, who reviewed and coded comments to identify ambiguities, contextual limitations, or suggestions for modification. These insights were then integrated with the quantitative results to guide decisions about revising or excluding EPAs between rounds.

Internal consistency of panel ratings was assessed using Cronbach’s alpha with 95% confidence intervals, a widely used reliability index in Delphi studies. Although interrater reliability indices, such as the intraclass correlation coefficient, can provide additional insights, they were not applied here, as the focus was on consistency of ratings across items rather than agreement between raters.

## 3 Results

### 3.1 Preliminary phase: development of EPA list

The draft EPA list was reviewed internally and finalized for evaluation in the Delphi rounds.

### 3.2 Second phase

Of the 45 invited experts, 21 (46.7%) completed both rounds of the Delphi survey. Most were from the Western region of Saudi Arabia (62%), with over 71% having more than 10 years of experience in neurology. However, 61.9% had fewer than 2 years of experience as residency program trainers, reflecting the recent expansion of the neurology program and the recruitment of new faculty members. The demographic characteristics of the participants are summarized in Table 1.

**Table 1:** Demographic characteristics of participants in the first round of the modified Delphi technique.

Demographic characteristics	*N*	(%)
City of practice		
Jeddah	7	33.3
Makkah	6	28.6
Riyadh	6	28.6
Tabuk	1	4.8
Abha	0	0.0
Dhahran	1	4.8
Years of experience as a neurologist		
3–5 years	3	14.3
5–10 years	3	14.3
> 10 years	15	71.4
Years of experience as a trainer in neurology program		
< 2 years	13	61.9
2–5 years	6	28.6
5–10 years	2	9.5
Status in neurology program		
Co-Program Director	1	4.8
Former Program Director	7	33.3
Program Director	3	14.3
Trainer in the Program	10	47.6

EPAs that met the predefined consensus threshold (mean score of ≥4.0, ≥80% agreement) were retained, while those that did not were either excluded or revised based on qualitative feedback. Thematic analysis of panelists’ comments identified three main themes guiding EPA revisions between rounds: (1) clarifying the scope of each EPA to align with general neurologist competencies (e.g., excluding subspecialty tasks such as muscle biopsy interpretation), (2) considering variability in resources and practice settings (e.g., access to genetic testing), and (3) distinguishing neurologist roles from allied health tasks (e.g., preventive counseling).

Consequently, 25 EPAs (69%) achieved consensus and were retained, 11 were excluded owing to insufficient relevance or agreement, and one was revised based on expert comments. Detailed ratings, standard deviations, and levels of disagreement are presented in Table 2.

**Table 2:** Suggested and modified EPAs across two rounds of the modified Delphi technique.

EPA	Round 1			Round 2		
	Mean	SD	Level of disagreement	Mean	SD	Level of disagreement
A. Clinical assessment						
A1. Obtaining a comprehensive history and performing a physical examination of patients present with neurological diseases	4.95	0.22	0	5	0	0
A2. Developing differential diagnosis based on patients with neurological clinical presentation	4.95	0.22	0	4.91	0.30	0
A3. Recognizing different comorbidities associated with neurological diseases (such as psychiatric, behavioral, systemic, and others)	4.81	0.40	0	4.76	0.44	0
A4. Composing an appropriate diagnostic approach for patients with neurological presentation	4.86	0.36	0	4.90	0.30	0
A5. Applying basic medical knowledge related to neurological diseases in daily medical practice (including: anatomy, physiology, immunology, pathology, genetics, and laboratory medicine)	4.38	0.74	14.29	4.57	0.51	0
A6. Interpreting laboratory tests relevant to the evaluation of patients with neurological diseases, including CSF analysis	4.86	0.36	0	4.95	0.22	0
A7. Interpreting relevant imaging studies (non-contrast CT, Contrast CT, basic vascular imaging, Brain and spinal cord MRI)	4.71	0.56	4.76	4.81	0.51	4.76
A8. Interpreting results of specialized neurological tests (EEG, NCS, and EMG) (Modified)	-			4.14	0.66	14.29
A9. Demonstrating expertise in utilizing genetic tests for neurological diseases	3.95	0.92	28.57			
A10. Interpreting results of specialized neurological tests (EEG, NCS and EMG, and Muscle biopsy)	4.10	0.94	23.81			
A11. Recognizing rare neurological presentations	4	1	23.81			
A12. Recognizing and triaging presentations of common pediatric neurological diseases (headache, stroke, epilepsy, and neuromuscular disease)	4.14	0.96	23.81			
B. Clinical management						
B1. Providing initial assessment, diagnosis, and management for patients with a range of acute and chronic neurological diseases	4.81	0.40	0	4.95	0.21	0
B2. Managing patients with neurological presentations in the ER, outpatient, inpatient, ICU, and chronic care facility	4.81	0.40	0	4.86	0.36	0
B3. Applying an appropriate management approach for patients with stable, chronic neurological conditions	4.57	0.51	0	4.52	0.60	4.76
B4. Applying an appropriate management approach for patients with uncertainty in the diagnosis and/or treatment of a neurological condition	4.29	0.78	23.81			
B5. Supporting adolescents/ young adults with neurological disease in the transition from the pediatric to adult care setting	4.14	0.96	23.80			
B6. Providing nutritional, behavioral, preventive, and non-pharmacological counseling for patients with neurological disease	4.14	0.96	28.57			
B7. Managing patients with neurological diseases in various circumstances (Ex. pre-conceptual period, during pregnancy, during fasting, perioperative period, during critical illnesses, infections, and malignancies)	4.52	0.60	4.76	4.43	0.68	9.5
B8. Applying medical knowledge that includes the mechanisms of action, the different forms, indications for usage, relative costs, risks, benefits, and the potential complications of medications commonly used in neurology	4.48	0.60	4.76	4.52	0.60	4.76
B9. Applying recommendations for management guidelines and evidence-based literature to the care of patients with neurological diseases	4.57	0.60	4.76	4.62	0.59	4.76
B10. Prescribing current and newly approved drugs in neurology practice	4.29	0.64	9.52	4.38	0.59	4.76
C. Clinical procedure						
C1. Demonstrating expertise in performing lumbar puncture	4.76	0.43	0	4.67	0.57	4.76
D. Transferable skills						
D1. Counseling patients and/or families regarding diagnosis and treatment plans for neurological diseases	4.62	0.5	0	4.71	0.64	9.52
D2. Implementing the principles of quality assurance and patient safety	4.43	0.75	14.29	4.71	0.56	4.76
D3. Developing a personal learning plan for future practice and ongoing professional development	4.38	0.59	4.76	4.52	0.51	0
D4. Participating in and/or leading educational or administrative activities	3.86	1.28	28.57			
D5. Delivering scholarly teaching to a variety of audiences, including peers, junior trainees, and/or other health professionals	4.24	0.89	23.81			
D6. Completing adequately written documentation for patient care	4.19	0.36	0	4.62	0.59	4.76
D7. Managing a long-term, structured, outpatient neurology clinic	4.71	0.46	0	4.52	0.60	4.76
D8. Critiquing and appraising current neurology literature	4.52	0.68	7.69	4.14	0.79	23.81
D9. Conducting research projects related to neurology	3.90	1.14	33.33			
D10. Demonstrating professional consultancy skills, utilizing resources, and considering other specialties	4.29	0.72	9.52	4.48	0.60	4.76
D11. Promoting health in response to society’s needs	4.10	1.04	23.81			
D12. Providing/recommending appropriate referrals to other health care providers necessary for adjunctive evaluation and/or management	4.48	0.68	0	4.52	0.60	4.76
D13. Providing neurology consultations to other specialties and providers	4.67	0.58	0	4.57	0.50	0
D14. Working with multidisciplinary teams to coordinate the care of patients with neurological diseases	4.5	0.60	4.76	4.57	0.60	4.76

The color indicating the high disagreement level/so EPAs were taken of from our final EPAs list.

The updated list of 26 EPAs was carried forward to the second round, which all 21 participants completed. Strong agreement was achieved across all EPAs (mean rating of ≥4 and ≥80% of panelists in agreement), resulting in a final set of 25 EPAs (Table 3).

**Table 3:** EPAs for the neurology training program in Saudi Arabia.

Domain	Saudi EPAs
Clinical assessment	1. Obtaining a comprehensive history and performing a physical examination of patients present with neurological diseases.
	2. Developing differential diagnosis based on the patient’s neurological clinical presentation.
	3. Recognizing different comorbidities associated with neurological diseases (such as psychiatric, behavioral, systemic, and others).
	4. Composing an appropriate diagnostic approach according to the patient’s neurological presentation.
	5. Applying basic medical knowledge related to neurological diseases in daily medical practice (including: anatomy, physiology, immunology, pathology, genetics, and laboratory medicine).
	6. Interpreting laboratory tests relevant to the evaluation of patients with neurological diseases, including CSF analysis and results.
	7. Interpreting relevant imaging studies (non-contrast CT, contrast CT, basic vascular imaging, brain and spinal cord MRI).
	8. Interpreting results of specialized neurological tests (EEG, NCS, and EMG).
Clinical management	1. Providing initial assessment, diagnosis, and management for patients with a range of acute and chronic neurological diseases.
	2. Managing patients with neurological presentation in the ER, outpatient, inpatient, ICU, and chronic care facility.
	3. Applying an appropriate management approach for patients with stable, chronic neurological conditions.
	4. Managing patients with neurological diseases in various circumstances (Ex. pre-conceptual period, during pregnancy, during fasting, perioperative period, during critical illnesses, infections, and malignancies).
	5. Applying medical knowledge that includes the mechanisms of action, the different forms, indications for usage, relative costs, risks, benefits, and the potential complications of medications commonly used in neurology.
	6. Applying recommendations for management guidelines and evidence-based literature to the care of patients with neurological diseases.
	7. Prescribing current and newly approved drugs in neurology practice.
Procedures	1. Performing lumbar puncture.
Transferable skills	1. Counseling patients and/or families regarding diagnosis and treatment plans for neurological diseases.
	2. Implementing the principles of quality assurance and patient safety.
	3. Developing a personal learning plan for future practice and ongoing professional development.
	4. Completing adequately written documentation for patient care.
	5. Managing a long-term, structured, outpatient neurology clinic.
	6. Demonstrating professional consultancy skills, utilizing resources, and considering other specialties.
	7. Providing/recommending appropriate referrals to other health care providers necessary for adjunctive evaluation and/or management.
	8. Providing neurology consultations to other specialties and providers.
	9. Working with multidisciplinary teams to coordinate the care of patients with neurological diseases.

Decisions were guided by both predefined quantitative criteria and qualitative input, ensuring a transparent audit trail. For example, one EPA initially combined electroencephalography (EEG), nerve conduction studies (NCS), electromyography (EMG), and muscle biopsy interpretation; removal of muscle biopsy interpretation, a subspecialty skill, improved consensus in round two.

### 3.3 Reliability assessment

The reliability of responses across both modified Delphi rounds was evaluated using Cronbach’s alpha. Cronbach’s alpha values were 0.95 in Round 1 and 0.91 in Round 2, indicating excellent internal consistency of expert ratings. These findings support the reliability and consistency of consensus decisions across both rounds.

## 4 Discussion

This study successfully developed and validated a set of 25 EPAs tailored to neurology residency programs in Saudi Arabia. The process followed a structured methodology involving expert consensus, using a modified Delphi technique to ensure content validity, contextual relevance, and alignment with international standards.

The initial development of 36 preliminary EPAs was grounded in a comprehensive review of existing neurology curricula from the SCFHS, Royal College of Physicians and Surgeons of Canada, and American Board of Psychiatry and Neurology. The categorization of EPAs into clinical assessment, clinical management, procedures, and transferable skills mirrors the core competencies expected of neurology residents globally, while adapting them to the Saudi training context.

Although most participating experts had extensive clinical experience, many had limited experience in supervising residency training. A modified Delphi method was employed, involving two survey rounds. While 71% of respondents had over 10 years of experience, 61.9% had fewer than 2 years of experience in residency training supervision. This likely reflects the recent expansion of neurology programs and the recruitment of new faculty members across Saudi Arabia. Nevertheless, the panel’s demographic diversity enriched the consensus process by ensuring a wide range of clinical and educational perspectives.

Several EPAs were excluded during the Delphi rounds because of perceived irrelevance or variability in practice across institutions, highlighting differences in neurology practice patterns within Saudi Arabia. One such example is EPA A9 (Table 2), related to a genetic disorder. Although recognition of the genetic background of many neurological disorders is increasing (28–30), genetic testing is limited to highly specialized centers. Our preliminary EPAs included the ability to perform appropriate genetic testing for neurological disorders. However, most adult neurology graduates are expected to practice in primary or secondary care centers, where such testing is typically unavailable. In routine clinical practice, patients with suspected genetic disorders are typically referred to specialized centers. Consequently, this EPA did not achieve a score of 4 owing to high variability in responses and was excluded from the pre-final list.

One modified EPA, originally covering the interpretation of EEG, NCS, EMG, and muscle biopsy (EPA A10), received mixed consensus. While experts agreed on the importance of EEG, NCS, and EMG for general neurologists, they considered muscle biopsy interpretation as a subspecialty task. Accordingly, the EPA was revised to exclude muscle biopsies. This modification improved consensus in the second round, achieving a mean score of 4.143 (SD = 0.655) and 86% agreement.

The distinction between reading and interpreting diagnostic tests has emerged as a key point. Although full interpretation may require subspecialty training (such as in epilepsy or neuromuscular disorders), the ability to understand and integrate test results into clinical decision-making remains essential for general neurology practice. EEG and neuromuscular rotations are mandatory components of various neurology programs (24, 26, 27), and specific competencies have been established for these rotations. Although EEG is an important diagnostic test in neurology, reading EEGs without supervision can be challenging owing to limited exposure and training (29). A prior study revealed that approximately 43% of neurology residents are unable to read EEGs independently (30). Educational activities related to EEG instruction vary widely across different programs (31), and the number of EEGs performed each month differs by training center (32). The availability of an epileptologist strongly influences the quality of EEG training. Therefore, a general neurologist is not expected to read an EEG independently but must be able to interpret its results. Similar principles apply to NCS and EMG tests, which require adequate exposure, usually obtained through focused subspecialty training. In our end-of-training EPAs, we emphasized the interpretation of EEG, NCS, and EMG (EPA A8) (Table 2) rather than their technical reading, as interpretation represents an essential competency for general neurologists.

Another debated domain was preventive care and counseling. While acknowledged as important, several respondents perceived this area as overlapping with the responsibilities of allied health professionals, such as nutritionists and health educators. Consequently, some EPAs related to preventive care did not reach consensus. Although preventive therapy and counseling are integral to managing neurological disorders, most respondents felt that these were not exclusive EPAs for neurologists, as they can be provided by other health specialists.

The final list of EPAs in adult neurology comprised 25 core activities representing essential skills that must be acquired by graduates of neurology programs for independent practice. The development of an educational program is a dynamic process designed to accommodate rapid changes in clinical practice. Competency-based curricula emphasize objective outcomes rather than fixed training durations (31), which can be challenging because educational programs have specific times for each rotation. Moreover, traditional curricula may not directly assess whether trainees can perform tasks unsupervised. The Saudi Board of Neurology has identified and mapped specific competencies for each training level (24). Applying an EPA-based curriculum helps integrate different competencies into clinical practice, ensuring that neurology graduates can be trusted in unsupervised settings. This concept may bridge the gap between theoretical knowledge and clinical practice (32), ultimately enhancing patient outcomes and safety.

EPAs offer several advantages over traditional competency-based frameworks. Although competencies define individual skills and knowledge, EPAs integrate them into coherent units of professional practice, thereby enabling entrustment decisions. This approach reflects the complexity of real-world clinical performance and enhances the validity of workplace-based assessments (33).

Implementing EPAs into neurology residency training programs requires a multistep process encompassing curricular mapping, faculty development, and assessment redesign. Each EPA should be aligned with existing competencies and mapped to the appropriate training year and supervision level, ranging from direct observation to independent practice and, ultimately, supervision of others. For example, early EPAs (taking a comprehensive patient history) may be expected of postgraduate year (PGY)-1 residents, whereas complex EPAs (such as interdisciplinary coordination) may apply to PGY-5 residents, who should be capable of supervising junior residents. A previously published national framework (23) has described an approach for implementing neurology EPAs, using the consolidated framework for implementing research as an organizing framework.

Standardizing the assessment of EPAs is a critical prerequisite for successful implementation. Transitioning to an EPA-based curriculum requires a paradigm shift from traditional numerical grading to entrustment decisions based on the level of supervision a trainee requires (31). This approach focuses on the readiness of the trainee to perform specific tasks independently rather than solely on knowledge acquisition. The assessment of EPAs can be effectively integrated into daily clinical practice through various strategies, including direct observation, structured knowledge testing, simulation-based evaluations, and both short- and long-term workplace assessments (31).

Faculty development is an essential step before implementing an EPA-based curriculum (34, 35) to ensure accurate entrustment decisions. Trainers must be equipped to observe EPA performance effectively and to provide constructive, actionable, and timely feedback in clinical settings (34, 35). Without adequate faculty preparation, the educational potential of EPAs may not be fully realized. Furthermore, following implementation, regular reviews and revisions of EPA-based programs are essential to accommodate ongoing changes in clinical practice, technology, and patient expectations.

Neurology training programs vary globally (36–38), and the generalizability of our results may be affected by the availability of different tests and facilities. In some countries lacking formal subspeciality training programs (39, 40), general neurologists are expected to manage complex cases and perform procedures not included in our EPA set. Moreover, although the validated EPAs in this study reflect the Saudi context, they may also inform EPA development in similar regions, particularly where subspecialty resources are limited.

Despite the methodological rigor of this study, it has some limitations. First, although efforts were made to recruit a nationally representative panel, most participants were from the Western region of Saudi Arabia, which may have limited the generalizability of the results across other regions with different training structures or resources. Second, although the panel included experienced neurologists, a substantial proportion had limited experience as formal trainers, which may have influenced their perspectives on supervision and trust. Third, a subset of the panel included early-career neurologists (3–5 years of experience), whose limited exposure to training supervision may have affected their perspective on the applicability of EPAs. Fourth, the study relied on expert consensus rather than empirical validation in clinical settings. The actual implementation and evaluation of EPAs in clinical settings, specifically, their impact on trainee performance, supervision dynamics, and patient care outcomes, were not assessed. Future studies are needed to evaluate how these EPAs function in practice and explore the inter-rater reliability of entrustment decisions. Finally, the exclusion of certain EPAs, particularly those related to pediatric neurology or advanced diagnostic testing, was based on contextual relevance and resource availability. Although appropriate for the Saudi setting, this may limit the applicability of the EPAs to other countries or institutions with a broader scope of practice.

In conclusion, this study successfully developed and validated a set of 25 EPAs tailored to neurology residency training in Saudi Arabia. These EPAs represent essential tasks that graduating neurologists should be able to perform independently and reliably in clinical settings. The finalized EPA framework provides a foundational step toward implementing a workplace-based, outcome-driven curriculum in neurology postgraduate education. By integrating EPAs into training programs, educators can better align assessments with real-world clinical responsibilities and support structured entrustment decisions.

This study contributes to the limited body of literature on region-specific EPA development in adult neurology training. Its regional relevance enhances its potential impact on national curriculum reform and serves as a model for similar initiatives across other specialties and countries with comparable training environments. However, the effective adoption of EPAs requires additional steps, including curricular mapping, faculty development, and the standardization of assessment tools aligned with supervision levels. Ongoing evaluation and refinement of EPAs are also crucial to ensure their continued relevance and applicability in evolving healthcare environments.

## Data Availability

The raw data supporting the conclusions of this article will be made available by the authors, without undue reservation.
